# High-Coherence, Physically Separable Dual-Frequency Fiber Laser Based on Bidirectional Dual-Path Ring Cavity

**DOI:** 10.3390/s26072171

**Published:** 2026-03-31

**Authors:** Shihuai Li, Baibing Ji, Feng Zhu, Yiyu Gan, Zichen Li, Qiao Wen

**Affiliations:** 1 State Key Laboratory of Radio Frequency Heterogeneous Integration, College of Physics and Optoelectronic Engineering, Shenzhen University, Shenzhen 518060, China; 2 Key Laboratory of Optoelectronic Devices and Systems of Ministry of Education and Guangdong Province, College of Physics and Optoelectronic Engineering, Shenzhen University, Shenzhen 518060, China

**Keywords:** single frequency, separable dual frequency, high-coherence beat frequency, bidirectional dual-path ring cavity

## Abstract

Dual-frequency lasers with narrow linewidth and high coherence serve as essential light sources for systems such as heterodyne detection, LiDAR, and precision interferometry. However, existing technologies cannot directly separate the two frequency components at MHz-scale differences, which remains a persistent bottleneck in this field. In this paper, we present a dual-frequency fiber laser based on a bidirectional dual-path ring cavity. The proposed laser supports flexible switching between single-frequency and dual-frequency operation while allowing straightforward physical separation of the two outputs via intrinsic beam routing. In single-frequency mode, the two beams exhibit Lorentzian linewidths of 1.1 kHz and 1.16 kHz, respectively. In dual-frequency operation, the laser produces a beat signal at 470 MHz with a 3-dB linewidth of 340.2 Hz and a signal-to-noise ratio (SNR) exceeding 70 dB. This dual-frequency fiber laser provides a novel and practical source for heterodyne detection and LiDAR-based measurement systems.

## 1. Introduction

Owing to their exceptional frequency stability, ultra-narrow linewidth, and wide-range frequency tunability, dual-frequency lasers offer significant advantages for applications demanding high measurement accuracy, strong resistance to external interference, high SNR, multi-parameter sensing capability, and operation in dynamic environments. At present, they play an indispensable and significant role in applications such as heterodyne interferometry [1,2,3], Doppler velocimetry [4,5,6,7], laser radar (LiDAR) detection [8,9,10,11], and absolute distance measurement [12,13,14]. This underscores the critical importance of developing high-performance dual-frequency lasers.

A fundamental requirement in systems like coherent detection is the physical separation of the two-constituent single-frequency beams, which serve as the reference and measurement arms, respectively. Currently, a major challenge for dual-frequency lasers lies precisely in this beam separation. For example, separating a single frequency from a 1550 nm dual-frequency laser with a 500 MHz difference would necessitate an optical filter with a bandwidth of approximately 3.96 pm. Achieving such a narrow filtering bandwidth remains highly challenging with current filter technology [15]. To address this issue, research efforts have primarily centered on dual-frequency lasers featuring large frequency separations (typically from GHz to THz), such as dual-wavelength single-mode lasers and Brillouin-scattering-based dual-frequency lasers [16,17,18,19]. However, these systems then require photodetectors with ultra-high response speeds to convert the optical beat signal, presenting another significant technological hurdle.

To achieve dual-frequency lasers with smaller frequency differences, techniques such as Zeeman birefringence dual-frequency lasers (MHz-level difference) and orthogonally polarized dual-frequency lasers (hundreds of MHz-level difference) have been extensively studied [20,21,22]. A key limitation of these birefringence-based approaches is that the two beams exhibit different polarization states, which degrades their mutual coherence. In heterodyne detection systems, this necessitates additional polarization control and alignment to optimize interference, thereby increasing system complexity and operational overhead.

Furthermore, related studies have shown that dual-frequency lasers with MHz-level frequency differences can be realized using microcavity structures [23,24] as well as frequency modulation techniques [25,26]. Notably, in microcavity-based systems, the two laser frequencies are generated within the same cavity and experience identical environmental perturbations, which leads to significantly more stable beat signals. On the other hand, frequency modulation methods offer greater flexibility in adjusting the frequency difference. However, each approach has its own limitations: microcavity structures suffer from relatively long response times and polarization mismatch issues, while frequency modulation methods are hindered by high insertion loss in the modulation devices and increased system complexity.

Additionally, in the field of high-end sensing and precision detection, integrating multiple operating modes into a single device has emerged as a pivotal trend. This approach not only greatly improves a system’s versatility across diverse application scenarios but also contributes to structural simplification, enhanced integration, and improved ease of use [27,28]. A notable example is the narrow-linewidth fiber laser with switchable single-frequency and dual-frequency operation. Such a system can deliver high-coherence, ultra-narrow-linewidth single-frequency output when needed, while also allowing rapid switching to a dual-frequency mode capable of generating high SNR beat frequencies. This flexibility enables “one device, multiple functions” across various applications—from high-resolution spectroscopy to coherent detection and beyond. By elevating overall equipment performance, this integrated design strategy paves a key technical pathway toward next-generation intelligent sensing systems tailored for complex operational environments.

To address these limitations, this paper presents a 1550 nm dual-frequency fiber laser based on a bidirectional dual-path ring cavity structure for the first time. In this design, the two-constituent single-frequency beams exist as independent counter-propagating modes, both sustained by the same gain medium within the cavity. This inherent characteristic enables their straightforward physical separation via simple beam splitting. The two single-frequency outputs exhibit Lorentzian linewidths of 1.1 kHz and 1.16 kHz, respectively. Their interference generates a beat signal at 470 MHz with a 3-dB linewidth of 340.2 Hz. Over a one-hour measurement period, the beat signal’s SNR remained stable at approximately 70 dB, confirming the high mutual coherence between the two beams.

## 2. Experimental Principle

**Figure 1:**
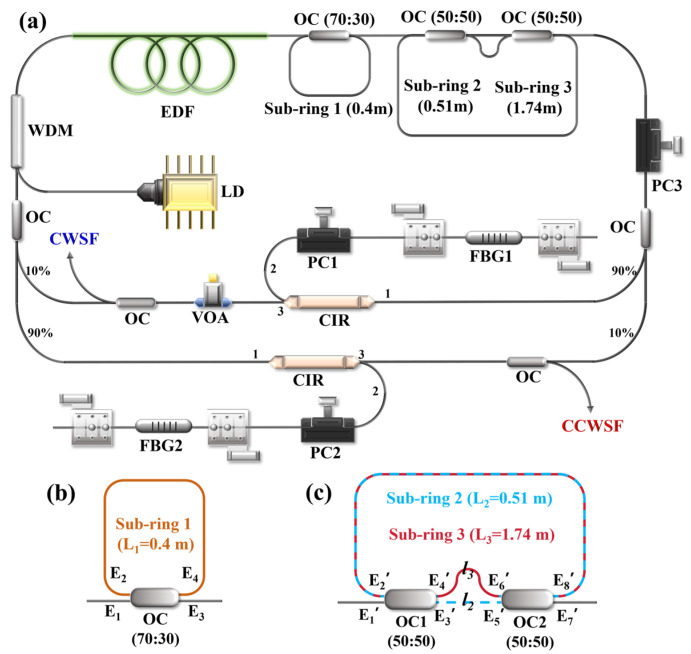
(**a**) Schematic of the 1550 nm bidirectional ring-cavity dual-frequency separable fiber laser (The figure indicates the ports 1, 2 and 3 of CIR. Light propagates within CIR in the direction from port 1 to port 2 and from port 2 to port 3); (**b**) Schematic of sub-ring 1; (**c**) Schematic of sub-rings 2 and 3.

The 1550 nm bidirectional ring cavity dual-frequency fiber laser built in this experiment is shown in Figure 1a. A 976 nm semiconductor laser was used to pump a 0.5-m-long erbium-doped fiber (EDF, Coherent, Santa Clara, CA, USA, 71 dB/m). The pump laser is coupled into the cavity through a wavelength division multiplexer (WDM), enabling the erbium-doped fiber to generate laser emission at 1550 nm.

Two 10:90 optocouplers (OCs) and two circulators (CIRs) are configured to form bidirectional paths. This setup enables the laser to propagate independently in the clockwise (CW) and counterclockwise (CCW) directions. The 90% output port of each OC is fused to port 1 of the corresponding CIR. This directs most of the circulating optical power from the common cavity (comprising the WDM and EDF) into the respective bidirectional branch. Port 2 of each CIR is respectively connected to the polarization controller (PC_1_ or PC_2_) and the fiber Bragg grating (FBG). The central wavelengths of the two FBGs are approximately 1550 nm, and the 3 dB bandwidth is 0.18 nm (approximately equal to 22.4 GHz). Two FBGs are fixed to the grooves of the high-precision displacement platform by tape and magnets. Since both FBGs used in this experiment were irradiated by femtosecond lasers, their inherent birefringence is usually 10^−7^ to 10^−6^, and they have a certain polarization-dependent loss (PDL) [29,30,31]. Therefore, mode competition within the cavity can be suppressed by managing the polarization-dependent loss. By adjusting the high-precision displacement platform, the fiber Bragg grating can be stretched, thereby changing the central wavelength of its reflection bandwidth. Port 3 of each CIR is fused to a 30:70 OC. This configuration feeds 70% of the light back into the cavity for oscillation and extracts 30% as the laser output. An additional PC_3_ is placed in the co-path optical path to simultaneously adjust the polarization states in both directions.

Additionally, FBG2 in the CCW path exhibits higher insertion loss than FBG1 in the CW path, a discrepancy likely arising from the femtosecond laser writing process. To compensate for this asymmetric cavity loss, a variable optical attenuator (VOA) was inserted after port 3 of the CW CIR. By adjusting the VOA, three distinct operating states can be selectively achieved: exclusive CW oscillation, exclusive CCW oscillation, or simultaneous bidirectional oscillation. With the VOA set to 0 dB attenuation, the lower intrinsic loss of the CW path grants it a decisive advantage in the intracavity gain competition, leading to CW-only operation. Conversely, when the VOA attenuation is maximized, the introduced loss in the CW path becomes dominant, thereby suppressing the CW laser and enabling CCW-only operation. By precisely tuning the VOA to equalize the net loss in both directions, stable bidirectional simultaneous oscillation is realized [32]. This active loss control is essential for reliably obtaining the simultaneous, stable operation of two single-frequency outputs from a single cavity, which defines the dual-frequency laser. Within the shared cavity section, three coupled sub-ring resonators are incorporated to constitute a high-finesse mode-selection filter. This filter simultaneously narrows the linewidths of both the CW and CCW propagating waves [33]. The principle of single-frequency selection is explained here for the CW direction; the same principle applies symmetrically to the CCW direction.

Based on the Jones matrix model of OC [34,35,36], the transmittance spectrum of Sub-ring 1 can be derived, as plotted in Figure 1b. The coupling equations of the OC relate the output optical fields at ports 3 and 4 to the input fields at ports 1 and 2, given as follows:

(1) $$\begin{array}{ccc} E_{3} & = & a\left(\sqrt{1-\gamma }E_{1}+i\sqrt{\gamma }E_{2}\right)\\ E_{4} & = & a\left(i\sqrt{\gamma }E_{1}+\sqrt{1-\gamma }E_{2}\right) \end{array}$$

where *γ* is the power coupling ratio, *a* is the transmission coefficient, *E_n_* denotes the complex optical field amplitude at port *n* (*n* = 1, 2, 3, 4). Light exiting port 4 propagates through a spliced fiber of length *L*_1_ and re-enters at port 2, leading to the following relationship:

(2) $$E_{2}=ke^{\left(-b+j\beta \right)L_{1}}E_{4}$$

where *b* is the transmission loss coefficient of the optical fiber, *k* is the splicing loss of the optical fiber. Here, *β* is the propagation constant of the fiber, given by β=2πnυ/c, where *υ* is the optical frequency satisfying the main cavity resonance condition, *c* = 3 × 10^8^ m/s is the speed of light in vacuum, and *n* = 1.468 is the effective refractive index of the fiber core.

Combining Equations (1) and (2), the relationship between the optical fields at port 3 and port 1 can be derived. The transmittance function for Sub-ring 1 is therefore given by:

(3) $$T_{1}={\left|\frac{E_{3}}{E_{1}}\right|}^{2}=\frac{a^{2}\left[\left(1-\gamma \right)-2ak\sqrt{1-\gamma }e^{-bL_{1}}\cos\left(\beta L_{1}\right)+a^{2}k^{2}e^{-2bL_{1}}\right]}{1-2ak\sqrt{1-\gamma }e^{-bL_{1}}\cos\left(\beta L_{1}\right)+a^{2}k^{2}\left(1-\gamma \right)e^{-2bL_{1}}}$$

Following the same methodology, the transmittance functions for Sub-ring 2 and Sub-ring 3 are derived, as plotted in Figure 1c and expressed in Equations (4) and (5), respectively:

(4) $$T_{2}={\left|\frac{E_{7}^{\prime }}{E_{1}^{\prime }}\right|}^{2}=\frac{a^{4}k^{2}(1-\gamma_{1})(1-\gamma_{2})e^{-2bl_{2}}}{1+2a^{2}k^{2}\sqrt{\gamma_{1}\gamma_{2}}e^{-bL_{2}}cos(\beta L_{2})+a^{4}k^{4}\gamma_{1}\gamma_{2}e^{-2bL_{2}}}$$

(5) $$T_{3}={\left|\frac{E_{7}^{\prime }}{E_{1}^{\prime }}\right|}^{2}=\frac{a^{4}k^{2}\gamma_{1}\gamma_{2}e^{-2bl_{3}}}{1-2a^{2}k^{2}\sqrt{\left(1-\gamma_{1})(1-\gamma_{2}\right)}e^{-bL_{3}}cos(\beta L_{3})+a^{4}k^{4}(1-\gamma_{1})(1-\gamma_{2})e^{-2bL_{3}}}$$

where *γ*_1_ and *γ*_2_ are the power coupling ratios the OC_1_ and OC_2_ respectively. *l*_2_ denotes the length of Sub-ring 2 from $E_{3}^{\prime }$ to $E_{5}^{\prime }$, *l*_3_ is the length of Sub-ring 3 from $E_{4}^{\prime }$ to $E_{6}^{\prime }$, *L*_2_ and *L*_3_ are the total length of Sub-ring 2 and Sub-ring 3.

Additionally, the 3 dB passband bandwidth of the sub-ring filter can be calculated as:

(6) $$\begin{array}{ccc} \Delta \nu_{3dB} & = & 2\left|v_{T_{\max}}-v_{T_{mid}}\right|\\ T_{mid} & = & \frac{1}{2}\left|T_{\max}+T_{\min}\right| \end{array}$$

where $\nu_{T_{\max}}$ and $\nu_{T_{mid}}$ are the optical frequencies at the maximum and half-maximum points of a single transmission peak, while *T_max_* and *T_min_* represent the peak transmission and the minimum transmission (background) of the resonance, and *T_mid_* is their arithmetic mean.

We simulated the filtering responses generated under various lengths and coupling ratios of three sub-rings and calculated the 3 dB linewidth of the final filtering response resulting from their superposition. To achieve single-frequency operation, the parameters were optimized such that the overall filter bandwidth was less than the cavity’s free spectral range (FSR). The total cavity length is ~7 m, yielding an FSR of ~29.19 MHz. The chosen parameters are: lengths of Sub-rings 1, 2, and 3 are 0.4 m, 0.51 m, and 1.74 m, respectively. The coupling ratio for Sub-ring 1 is 70%; and the coupling ratios for OC_1_ and OC_2_ in Sub-rings 2 and 3 are both 50%. The resultant composite filter has a full width at half maximum (FWHM) of 28.66 MHz. Since this is narrower than the FSR, it effectively suppresses adjacent longitudinal modes, satisfying a key requirement for single-frequency operation.

The FSR of Sub-rings 1, 2, and 3 is 511 MHz, 401 MHz, and 117 MHz respectively. Each FSR is given by:

(7) $$FSR=\frac{c}{nL}$$

**Figure 2:**
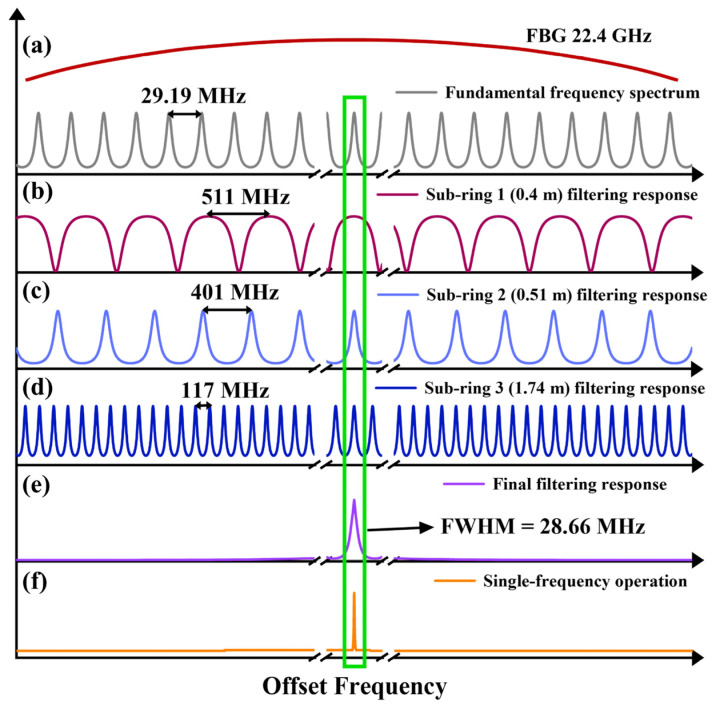
Schematic of single-frequency operation in one direction (The green box indicates the corresponding relationship between the single longitudinal mode that has been selected and multiple sub-ring filtering peaks.). (**a**) FBG reflection spectrum and the fundamental cavity modes; (**b**–**d**) Filtering responses of sub-rings 1, 2, and 3, respectively; (**e**) Overall filtering response resulting from the cascade of (**b**–**d**); (**f**) Selected single-frequency laser output.

Based on the Vernier effect in a compound cavity [37], the effective FSR of the laser corresponds to the least common multiple of the individual sub-ring FSRs. This condition is met when:

(8) $$FSR_{eff}=n_{1}FSR_{Sub\text{-}ring1}=n_{2}FSR_{Sub\text{-}ring2}=n_{3}FSR_{Sub\text{-}ring3}$$

where *n*_1_, *n*_2_ and *n*_3_ are positive integers. This yields an effective FSR for the composite cavity that is significantly larger than 22.4 GHz (the bandwidth of the FBG). Consequently, within the reflection band of a single FBG, only one longitudinal mode of the composite cavity can oscillate, enabling single-frequency operation. A schematic illustrating the mode selection by the three coupled sub-rings is presented in Figure 2.

## 3. Single-Frequency Characteristic

**Figure 3:**
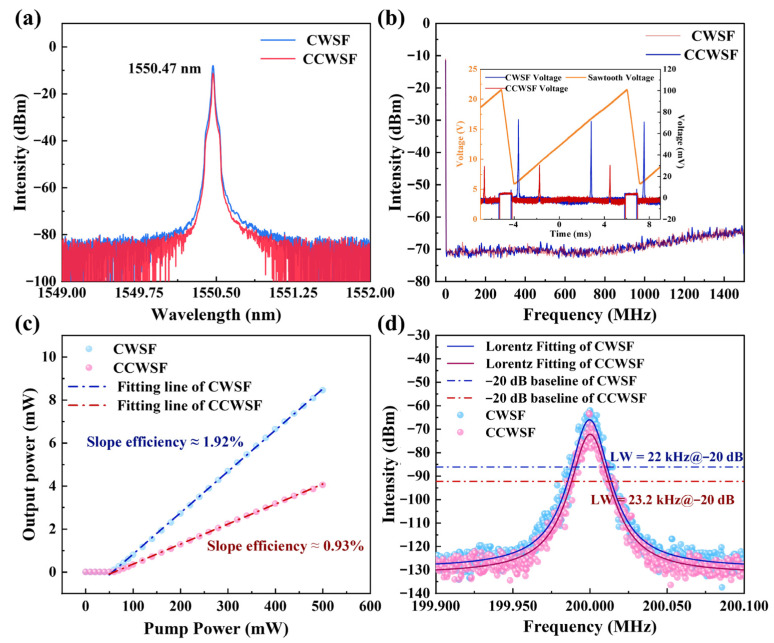
Characteristics of the single-frequency laser when oscillating separately in the CW and CCW directions. (**a**) Optical spectrum; (**b**) RF spectrum and SFPI signal (inset) within a 1.5 GHz range; (**c**) Slope efficiency; (**d**) Linewidth.

Figure 3a shows the optical spectra of the laser when operating in each direction separately, measured with an optical spectrum analyzer (OSA, YOKOGAWA AQ6370D, Tokyo, Japan). First, the pump power was set to 200 mW and the VOA attenuation was minimized (0 dB). Under this condition, only the clockwise single-frequency (CW-SF) laser oscillates. Its spectrum is plotted as the blue curve in Figure 3a. Subsequently, the VOA attenuation was increased to its maximum. This made the loss in the CW path significantly exceed that in the CCW path, thereby suppressing the CW-SF laser and allowing only the counterclockwise single-frequency (CCW-SF) laser to oscillate. Its spectrum is shown as the red curve in Figure 3a. From Figure 3a, it can be seen that the spectrum intensity of CW-SF is higher than that of CCW-SF. This is due to the inconsistent FBG losses in the two paths. When the pump power is the same at 200 mW, the output laser powers when they oscillate independently are different, which will lead to inconsistent shot noise and thermal noise [38,39,40]. Therefore, the noise intensity of CW-SF is higher than that of CCW-SF. The inset of Figure 3b displays the corresponding signals measured by a scanning Fabry–Pérot interferometer (SFPI, Thorlabs, Newton, NJ, USA). For each direction, only one set of interference peaks is observed over one SFPI scan period (blue curve for CW-SF, red for CCW-SF), confirming single-frequency operation in both cases. To further verify the single-frequency nature, the output from each laser was separately detected by a high-speed photodetector (PD, EOT ET-500F, Coherent, Santa Clara, CA, USA). The RF output of the PD was then analyzed by an RF spectrum analyzer (Rigol DSA815, Beijing, China). As shown in Figure 3b, the RF spectra for both lasers show no measurable beat frequencies or spurious signals within the 1.5 GHz range, providing further evidence of stable single-frequency operation for both the CW-SF and CCW-SF outputs.

The slope efficiency was characterized under two operating conditions: CW-SF only and CCW-SF only as plotted in Figure 3c. The data yield slope efficiencies of 1.92% for the CW-SF laser (blue curve) and 0.93% for the CCW-SF laser (red curve). The higher slope efficiency of the CW-SF laser is consistent with the lower intracavity loss in the clockwise direction, primarily attributed to the lower insertion loss of FBG1 compared to FBG2. The linewidth of each single-frequency output was measured separately using a delayed self-heterodyne (DSH) interferometer [41]. This method was chosen for its high resolution and immunity to low-frequency noise, as it does not require an external reference laser. Figure 3d shows the measured beat frequencies from the DSH interferometer for the CW-SF (blue curve) and CCW-SF (red curve) outputs. The measured linewidths at the −20 dB points are 22 kHz and 23.2 kHz, respectively. Assuming a Lorentzian lineshape, the corresponding 3-dB linewidths (FWHM) are calculated to be 1.1 kHz and 1.16 kHz. The achieved narrow linewidth can be understood through the Schawlow–Townes formula, which states that the fundamental laser linewidth is inversely proportional to the photon lifetime and the cavity quality factor [42]. A key approach to reducing the linewidth is to extend the photon lifetime within the resonator [43,44]. In our design, this is accomplished by coupling multiple sub-rings to form a compound cavity, which effectively increases the photon lifetime and thus suppresses the phase noise, leading to ~1 kHz linewidths observed. Furthermore, the noise level of the CW-SF power density spectrum is slightly higher than that of the CCW-SF. This is caused by the sidebands generated by the delay heterodyne system. The intensity of the sidebands is proportional to the incident light power [45].

## 4. Dual-Frequency Characteristics

The dual-frequency operation of the laser was then investigated. To characterize this state, the outputs from both directions were combined using a 3-dB (50:50) fiber coupler to generate an optical beat signal. This combined signal was then split by a second 3-dB coupler. One output of this splitter was sent to a SFPI, and the other to an OSA. The VOA was carefully adjusted to balance the intracavity losses, enabling stable simultaneous oscillation in both directions. Under these conditions, with the pump power set to 400 mW, two distinct spectral peaks were observed on the OSA, confirming dual-frequency operation.

**Figure 4:**
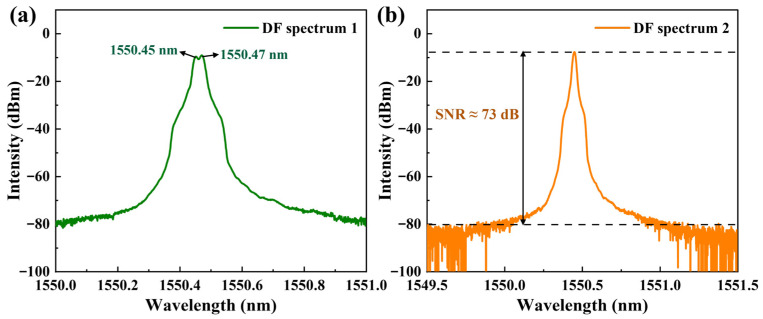
Optical spectra under bidirectional simultaneous oscillation. (**a**) Spectra of the two counter-propagating beams; (**b**) Spectra of the two beams, aligned at the same wavelength (coincident spectra).

As shown in Figure 4a, the central wavelengths are 1550.45 nm for the CCW-SF laser and 1550.47 nm for the CW-SF laser. The VOA was then finely adjusted to balance the intracavity losses, enabling stable simultaneous oscillation of both single-frequency lasers with nearly equal output power. Subsequently, the Bragg wavelengths of the two FBGs were tuned (e.g., by stretching) to align the output spectra to the same position, as shown in Figure 4b. Given the 0.02 nm resolution limit of OSA, the appearance of a single peak indicated that the two laser wavelengths were matched within this resolution. Precise wavelength alignment is necessary to observe the beat frequency. The measurement range of the RF spectrum analyzer is 0–1.5 GHz, which corresponds to a wavelength span of only about 0.012 nm in the 1550 nm band. Therefore, the laser wavelengths must be brought to within this small difference for the beat frequency to fall within the detectable range. Finally, as seen in Figure 4b, the SNR of the overlapped spectrum under dual-frequency operation reaches approximately 73 dB.

**Figure 5:**
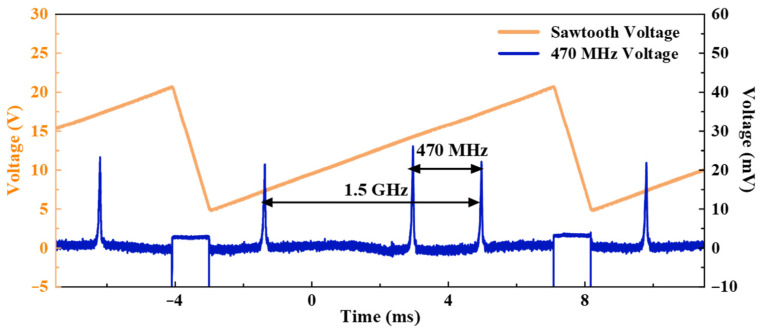
SFPI trace confirming the dual-frequency operation with a 470 MHz frequency separation.

**Figure 6:**
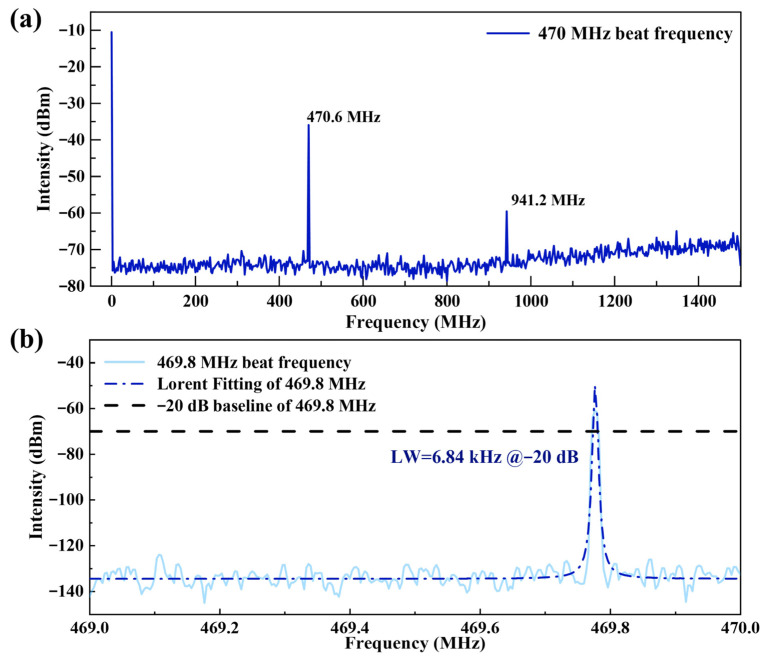
(**a**) RF spectrum of the 470 MHz beat frequency; (**b**) Linewidth measurement of the beat frequency.

Figure 5 shows the trace from the SFPI as displayed on an oscilloscope (Tektronix, Shanghai, China). Within one scan cycle (driven by a sawtooth voltage), three distinct peaks are observed. The two peaks separated by 1.5 GHz correspond to two successive longitudinal modes of the CCW-SF laser, separated by the FSR of the SFPI. The second peak, offset by approximately 470 MHz from one of these CCW-SF peaks, corresponds to the CW-SF laser signal. The beat frequency generated by the interference of the CW-SF and CCW-SF lasers was measured directly using an RF spectrum analyzer, as shown in Figure 6a. The resulting RF spectrum, plotted over a 1.5 GHz span, reveals two distinct peaks at 470.6 MHz and 941.2 MHz. Since only two optical frequencies (CW-SF and CCW-SF) were present, the expected signal is a single beat frequency at their difference frequency. Therefore, the presence of the 941.2 MHz peak is attributed to the nonlinear response of the photodetector. Figure 6b shows the linewidth of the beat frequency signal at −20 dB. Assuming a Lorentzian lineshape, the corresponding FWHM is calculated to be 340.2 Hz.

**Figure 7:**
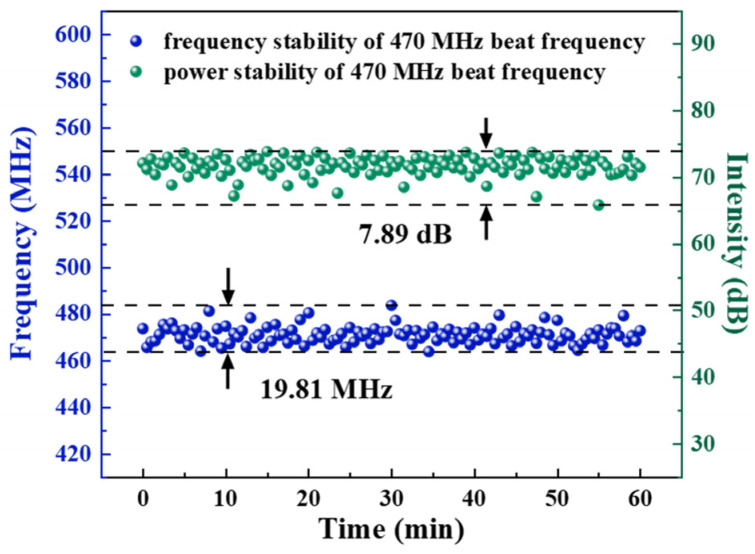
Long-term frequency and SNR stability.

Figure 7 illustrates the frequency stability and SNR stability of the 470 MHz beat frequency. During a one-hour measurement period, the 470 MHz beat signal exhibited a peak-to-peak frequency variation of approximately 19.81 MHz (±9.905 MHz). This fluctuation can be attributed to environmental factors. Fluctuations in ambient temperature and mechanical vibrations induce changes in the fiber refractive index. This, in turn, alters the resonant peak of the sub-ring filter, leading to a shift in the relative frequency between the CW-SF and CCW-SF lasers and thus the beat frequency. Furthermore, with the background noise floor of the RF spectrum analyzer at approximately −135 dBm, the SNR of the 470 MHz beat frequency remained stable at around 70 dB over the one-hour measurement. The observed variation in the SNR was 7.89 dB. Since this laser has a common optical path section, when these two bidirectional lasers encounter external interference in the common path section, the interference has the same effect on both lasers. This is reflected as synchronous changes in the frequency domain, so their frequency difference still remains equivalent to no change. This characteristic enables this laser to maintain good frequency difference stability even without packaging, without constant temperature control, and without frequency stabilization measures. However, it must be acknowledged that the changes caused by external interference to this laser are very obvious. If the laser can be encapsulated or a frequency stabilization device is added [46,47,48], the frequency difference stability will be further improved. Under these conditions, a dual-frequency laser with a 470 MHz frequency difference and the aforementioned characteristics has been successfully realized. The 470 MHz frequency difference is determined by the frequencies of the two single-longitudinal modes in the two-path single-frequency laser. Although the laser has a common optical path section and both directions share the same sub-ring structure for filtering, the filtering response produced by the sub-rings varies due to the different optical modes of the two paths. Additionally, the optical paths in the two branches are not exactly the same, so ultimately two different single-longitudinal modes with different frequencies will be selected separately, resulting in a 470 MHz frequency difference. This frequency difference corresponds to a wavelength difference of approximately 3.76 pm. This laser source directly emits two single-frequency beams with a 470 MHz separation. This built-in frequency difference eliminates the need for external, challenging-to-implement narrowband filters with picometer-scale selectivity.

## 5. Conclusions

In summary, we have demonstrated a 1550 nm dual-frequency fiber laser based on a bidirectional, dual-path ring cavity. In single-frequency operation, the measured 3-dB linewidths (Lorentzian fit) are 1.1 kHz for the CW output and 1.16 kHz for the CCW output. When both paths oscillate simultaneously in the dual-frequency state, a stable beat frequency at 470 MHz is generated. This beat frequency exhibits a high SNR of up to 70 dB and a narrow 3-dB linewidth of 340.2 Hz. As no active frequency stabilization was implemented, the beat frequency exhibited a frequency jitter of approximately ±9.905 MHz over a one-hour measurement period. The laser provides switchable operation between stable single-frequency and dual-frequency states. In the dual-frequency mode, it directly delivers two spectrally distinct optical carriers with a high-SNR radio-frequency beat signal inherent to their combination. This work provides a promising laser source for applications requiring a stable dual-frequency output, such as heterodyne detection systems and LiDAR-based measurements.

## Data Availability

The original contributions presented in this study are included in the article. Further inquiries can be directed to the corresponding author.

## Abbreviations

The following abbreviations are used in this manuscript:

SNR	Signal-to-noise Ratio
FBG	Fiber Bragg grating
PDL	Polarization-dependent loss
OC	Optocoupler
EDF	Erbium-doped fiber
WDM	Wavelength division multiplexer
CIR	Circulator
CW	Clockwise
CCW	Counterclockwise
CW-SF	Clockwise single frequency
CCW-SF	Counterclockwise single frequency
PC	Polarization controller
VOA	Variable optical attenuator
FSR	Free spectral range
FWHM	Full width at half maximum
SFPI	Scanning Fabry–Pérot interferometer
DSH	Delayed self-heterodyne
